# Characterization of the spore-forming *Bacillus cereus sensu lato* group and *Clostridium perfringens* bacteria isolated from the Australian dairy farm environment

**DOI:** 10.1186/s12866-015-0377-9

**Published:** 2015-02-19

**Authors:** Paul Dréan, Catherine M McAuley, Sean C Moore, Narelle Fegan, Edward M Fox

**Affiliations:** CSIRO Food and Nutrition, 671 Sneydes Road, Werribee, VIC 3030 Australia

**Keywords:** *Bacillus cereus sensu lato*, *Clostridium perfringens*, Dairy farm, PFGE, Toxin

## Abstract

**Background:**

The *Bacillus cereus sensu lato* group and *Clostridium perfringens* are spore-forming bacteria often associated with food spoilage and which can cause emetic and diarrheal syndromes in humans and ruminants. This study characterised the phenotypes and genotypes of 50 *Bacillus cereus s. l.* isolates and 26 *Clostridium perfringens* isolates from dairy farms environments in Victoria, Australia.

**Results:**

Five of the seven *B. cereus s. l.* species were isolated, and analysis of the population diversity using Pulsed-Field Gel Electrophoresis (PFGE) suggested that the populations are largely distinct to each farm. Enterotoxin production by representative isolates of each *B. cereus s. l.* species identified was typically found to be reduced in milk, compared with broth. Among the *C. perfringens* isolates, only two different toxin types were identified, type A and D. Bovine and ovine farms harbored only type A whereas both type A and D were found on two of the three caprine farms.

**Conclusions:**

This study showed that the *B. cereus s. l.* populations on the sampled farms exhibit a broad diversity in both species and genotypes. The risk of toxin-induced diarrheal illness through consumption of contaminated milk may be limited, in comparison with other food matrices. Type A strains of *C. perfringens* were the most abundant on dairy farms in Victoria, however type D may be of concern on caprine farms as it can cause enterotoxemia in goats.

**Electronic supplementary material:**

The online version of this article (doi:10.1186/s12866-015-0377-9) contains supplementary material, which is available to authorized users.

## Background

The *Bacillus cereus s. l.* species are Gram-positive, facultative anaerobic, spore-forming rods. As soil-associated ubiquitous organisms, *B. cereus s. l.* are commonly found in food products where they can cause spoilage [1]. In addition, pathogenic strains can be responsible for two different types of foodborne illness in humans. Diarrheal disease has been associated with three toxins: a single protein named cytotoxin K (CytK), and two heat-labile protein complexes: hemolysin BL (Hbl) and non-hemolytic enterotoxin (Nhe). Emetic syndrome is caused by the heat-resistant toxin cereulide. Emetic syndrome is usually associated with starchy food, such as rice or pasta, whereas diarrheal syndrome is caused by consumption of contaminated sauces, vegetables or dairy products [2]. The incidence of *Bacillus cereus* on dairy farms has been extensively studied, particularly in Scandinavia and the Netherlands [3,4].

The taxonomy of *B. cereus s. l.* is complex and has been regularly updated by new findings. To date, seven members have been described: *Bacillus cereus sensu stricto*, *Bacillus mycoides*, *Bacillus pseudomycoides, Bacillus thuringiensis*, *Bacillus weihenstephanensis*, *Bacillus anthracis* and most recently *Bacillus cytotoxicus* [5]*.* These strains share a highly conserved genome, and their 16S rRNA gene sequences show high levels of similarity [6]. Moreover, horizontal transfer of plasmid-carried genes is widespread among *B. cereus s. l.* strains [7]. These features led studies to question the relevance of the taxonomic segregation of *B. cereus s. l.* species on the basis of genetic closeness [8,9]. However, it is still possible to discriminate the *B. cereus* group strains, on the basis of various phenotypical traits. *B. mycoides* and *B. pseudomycoides* exhibit a typical rhizoid growth whereas the rest of the species have round to irregular colonies [10]. *B. mycoides* strains are able to grow at 8°C but not at 40°C. *B. weihenstephanensis* strains are psychrotolerant and can thus grow at temperatures as low as 7°C but not higher than 43°C [11]. On the contrary, *B. cytotoxicus* strains have a growth temperature range of 20°C to 50°C [5]. *B. thuringiensis* strain produce parasporal toxin crystals with insecticidal properties [12]*.* Finally, *B. anthracis* strains are non-hemolytic on Sheep Blood Agar (SBA) plates [13]*.*

*Clostridium perfringens* is a Gram-positive, anaerobic, spore-forming bacterium commonly found in the environment and in animal intestinal tracks. It is also a human pathogen and has been identified as the second most common bacterial source of foodborne illness in the United States [14]. *C. perfringens* strains are classified into toxigenic types A, B, C, D and E, based on the combination of four different toxins that they can produce (alpha-, beta-, epsilon- and iota-toxins). Moreover, the *C. perfringens* strains can also produce a diversity of other toxins, such as the beta2 enterotoxin and *Clostridium perfringens* enterotoxin (CPE).

The isolates in this study were obtained from samples from various sources (e.g., soil, water, faeces, animal feed, milk filter and raw milk) collected on a mixed group of bovine, caprine and ovine dairy farms in Victoria, Australia [15]. The diversity of *B. cereus s. l.* isolates was evaluated, in terms of taxonomy and toxin production potential at different temperatures in non-agitated reconstituted milk and broth. Molecular ecology of the *B. cereus s. l.* population was investigated using PFGE. *C. perfringens* isolates were subtyped using a toxin-gene-targeted multiplex PCR method. This study provided extensive data about the diversity of the on-farm populations of *Bacillus cereus s. l.* and *C. perfringens* in Victoria, Australia. The results regarding the growth conditions and toxin production potential of these pathogens provides information on the potential associated public health risk, and for the design of tailored bacterial control methods on the Victorian dairy farms.

## Results

### Identification of *Bacillus cereus s. l.* species

A total of 50 isolates were obtained from soil, faeces, feed (grain), raw milk and milk filter samples (27 samples total) collected on seven dairy farms. Where possible, two *B. cereus s.l.* isolates were taken from each sample to yield a total of 50 isolates from 27 positive samples (i.e. 4 samples only yielded a single, confirmed *B. cereus s.l.* isolate).

All the isolates exhibited hemolytic activity on SBA plates and were able to grow at a temperature of 10°C, indicating that no *B. anthracis* (non-hemolytic) or *B. cytotoxicus* (minimum growth temperature of 20°C) species were among the isolate pool.

**Figure 1:**
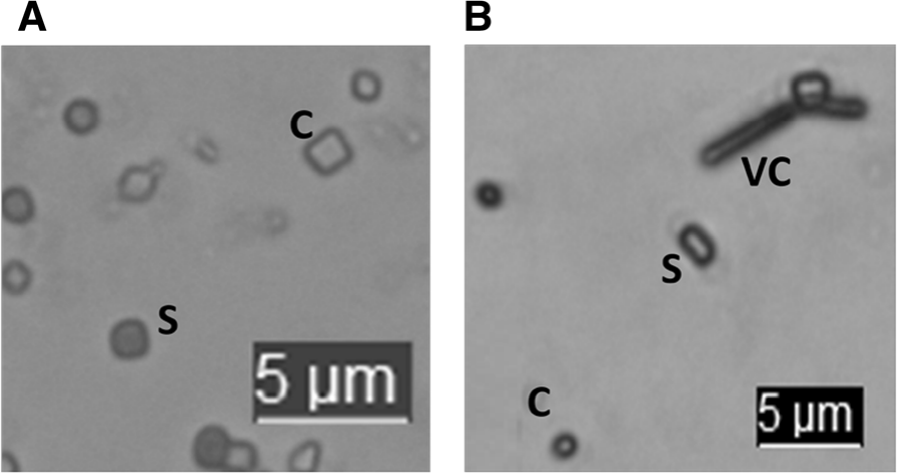
**Toxin crystal microscopy of representative** ***Bacillus thuringiensis*** **isolates.** C, toxin crystal; S, spore; VC, vegetative cell. **A**, bipyramidal toxin crystals produced by B13-014; **B**, round toxin crystals produced by Bc13-016.

Fourteen isolates could not grow at 8°C, identifying them as *B. cereus s. s.*, *B. thuringiensis*, or *B. pseudomycoides*. One of these exhibited a rhizoid colony morphology, and was classified as *B. pseudomycoides*. Of the remaining 13 isolates, three produced bipyramidal or round toxin crystals on sporulation, and were identified as *B. thuringiensis* (Figure 1). The remaining 10 isolates that were unable to grow at 8°C were classified as *B. cereus s. s.*

Another five isolates were incapable of growth at 40°C, implicating them as either *B. mycoides* or *B. weihenstephanensis*. Colony morphology was then used to differentiate these into *B. mycoides* (*n* = 3) showing rhizoid growth, and *B. weihenstephanensis*, with round to irregular colonies (*n* = 2).

The remaining 31 isolates were capable of growth at both 8°C and 40°C, exhibited a round to irregular colony morphology, and were implicated as either *B. cereus s.s* or *B. thuringiensis*. One of these isolates produced toxin crystals and was designated as *B. thuringiensis*; the other 30 isolates were identified as *B. cereus s. s.*

### Growth profiles of *B cereus s. l.* species at low temperatures

**Figure 2:**
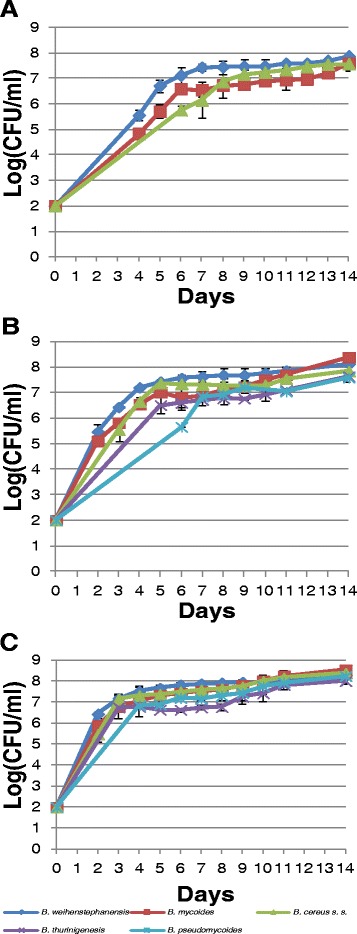
**Growth profiles of representative** ***B. cereus s. l.*** **species at low temperatures.** Growth was measured at 8^o^C **(A)**, 10^o^C **(B)**, and 12^o^C **(C)**, over a 14 day period.

The growth profile of one representative strain from each *B. cereus s. l.* species identified in this study (i.e. *B. cereus s. s.*, Bc14-005; *B. thuringiensis*, Bc13-016; *B. weihenstephanensis*, Bc14-026; *B. mycoides*, Bc14-009; and *B. pseudomycoides*, Bc14-006) is shown in Figure 2. At 8°C the *B. weihenstephanensis* isolate reached exponential and stationary phase in the shortest times, with an average cell density of 5.55 ± 0.2 log(CFU/ml) measured on day 4. This was followed by the *B. mycoides* isolate, with an average cell density of 4.83 ± 0.09 log(CFU/ml), also measured on day 4. The *B. cereus s. s.* isolate reached numbers of 5.75 ± 0.17 log(CFU/ml) by day 6, at which time the representative isolates of both other species has reached stationary growth phase. Neither the *B. thuringiensis* nor *B. pseudomycoides* representative isolates grew at 8°C.

The *B. weihenstephanensis* isolate also grew to higher cell densities on days 2, 3 and 4 at 10°C, when compared with the other 4 species. All representative isolates, with the exception of the *B. pseudomycoides* isolate, reached stationary stage after approximately 5 days at 10°C; this took approximately 7 days for the *B. pseudomycoides* isolate*.*

At 12°C, highest cell densities after 2 days were observed for *B. weihenstephanensis.* Cell growth plateaued after 3 days (all except *B. pseudomycoides*) or 4 days (for the *B. pseudomycoides* isolate).

### Genotypic diversity of *B. cereus s. l.*

**Figure 3:**
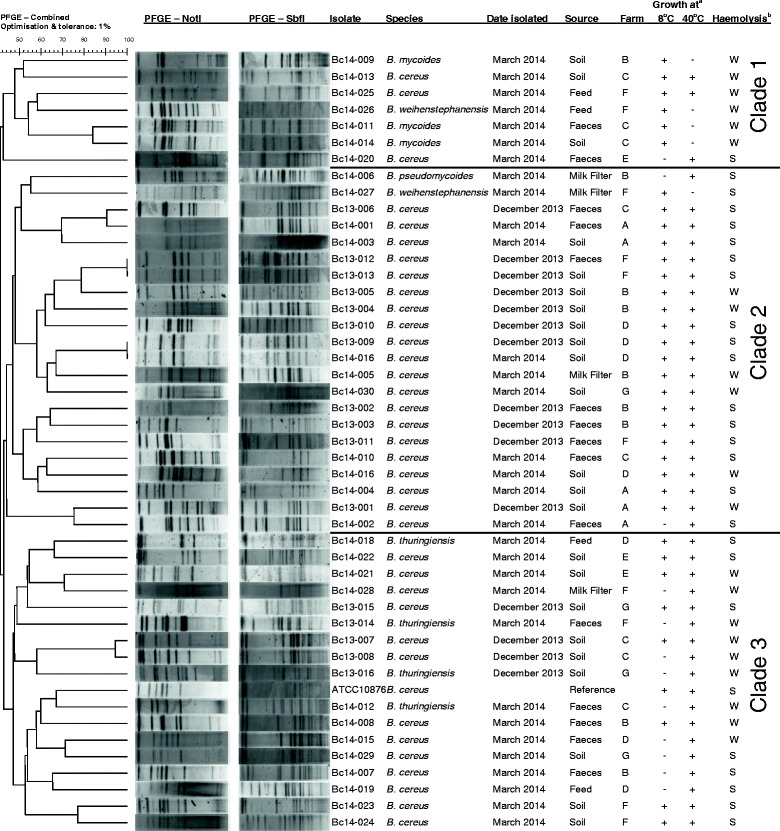
**Dendogram of the PFGE profiles of the** ***B. cereus*** **group isolates, using combined** ***Not*** **I and** ***Sbf*** **I restriction analysis.** ^a^+, growth; -, no growth. ^b^S, strong, W, weak.

PFGE subtyping was utilised to understand the population diversity in samples, across each farm environment, and comparatively across all farms (Figure 3). Two isolates were taken from each positive sample to examine strain diversity among samples. Comparison of both isolates from each sample identified the same strain in only four of the samples; in all other samples, where two isolates were obtained, they were identified as different strains. Where indistinguishable PFGE profiles (pulsotypes) were obtained for both isolates from the same sample, one of these isolates was removed from the study (this identified 4 isolates as duplicated). This resulted in the speciation of the remaining 46 isolates as: 36 *B. cereus s. s.*; four *B. thuringiensis*; three *B. mycoides*; two *B. weihenstephanensis*; and one *B. pseudomycoides. B. cereus s. s.* was identified on all 7 farms, *B. thuringiensis* on 4 farms, and *B. mycoides* on Farms B and C. Both *B. weihenstephanensis* strains were recovered from Farm F, and the *B. pseudomycoides* isolate was from Farm B.

Population analysis of the 46 remaining isolates yielded 44 distinct pulsotypes; two of these pulsotypes were identified in multiple samples (each of these strains were identified in two separate samples). A pulsotype was shared by *B. cereus s. s.* isolates Bc13-013 (isolated from soil) and Bc13-012 (isolated from a faecal sample); both samples were from Farm F, taken on the first visit (December 2013). The other pair of isolates indistinguishable by PFGE were Bc13-009 and Bc14-016, isolated from soil samples collected from Farm D in December 2013 and again in March 2014.

No pulsotypes identified in this study were shared across multiple Farms, and a diversity of between three and nine unique pulsotypes were identified on individual farms. The PFGE profiles clustered in 3 major groups: Clades 1, 2 and 3. Clade 1 comprised 7 isolates; all the *B. mycoides* isolates clustered together in this group, together with one *B. weihenstephanensis* isolate. The rest of the isolates were *B. cereus* isolates. Eighty-six percent of members of this grouping were psychrotrophic and weakly hemolytic; only Bc14-020 (showing the lowest similarity among clade 1 isolates) did not grow at 8°C, and was also strogly hemolytic. Clade 2 included 22 isolates: one *B. pseudomycoides*, one *B. weihenstephanensis* and 20 *B. cereus s. s.* isolates. Finally, clade 3 comprised 18 isolates, along with the *B. cereus* reference strain ATCC 10876. All the *B. thuringiensis* strains clustered in this last group. The remaining isolates were *B. cereus s. s.*. This group exhibited a broader phenotypic diversity, with 50% of strains capable of growth at 8°C. Similarly, 50% of strains displayed weakly hemolytic phenotypes.

### Production of enterotoxins by *Bacillus cereus s. l.* isolates

Production of enterotoxins Nhe and Hbl was greatest at 25°C, for all 5 species tested (Table 1). All five representative isolates produced Nhe enterotoxin in TSBYE after 48 h, however the *B. weihenstephanensis* representative isolate (Bc14-026) was the only isolate not to produce detectable Nhe after 24 h in TSBYE at this temperature. The *B. mycoides* Bc14-009 isolate was the only isolate to produce detectable Hbl enterotoxin under these conditions, after 48 h. With the exception of *B. weihenstephanensis*, all isolates produced higher amounts of Nhe in TSBYE at 25°C, when compared with production in 10% RSM. No detectable enterotoxin was produced by the *B. pseudomycoides* isolate in 10% RSM at 25°C, in contrast to strong Nhe production measured in TSBYE.

**Table 1:** **Toxin production by representative isolates of each** ***B. cereus s. l.*** **species identified in this study, under various combinations of temperature and growth medium**

		**12°C**				**25°C**				**37°C**			
		**10% RSM**		**TSBYE**		**10% RSM**		**TSBYE**		**10% RSM**		**TSBYE**	
		**Nhe**	**Hbl**	**Nhe**	**Hbl**	**Nhe**	**Hbl**	**Nhe**	**Hbl**	**Nhe**	**Hbl**	**Nhe**	**Hbl**
Bc14-005	24 h	NT	NT	NT	NT	++	-	+++	-	+	-	+++	-
*B. cereus s. s.*	48 h	+	-	-	-	++	-	+++	-	+	-	+++	-
Bc14-006	24 h	NT	NT	NT	NT	-	-	++	-	-	-	+	-
*B. pseudomycoides*	48 h	-	-	-	-	-	-	+++	-	-	-	+	-
Bc14-009	24 h	NT	NT	NT	NT	+	-	+++	-	-	-	+	-
*B. mycoides*	48 h	+	-	+	-	+	-	+++	+	-	-	+	-
Bc14-026	24 h	NT	NT	NT	NT	-	-	-	-	-	-	-	-
*B. weihenstephanensis*	48 h	-	-	-	-	+	-	+	-	-	-	-	-
Bc13-016	24 h	NT	NT	NT	NT	++	-	+++	-	+	-	++	+
*B. thuringiensis*	48 h	+	-	-	-	+	-	+++	-	++	-	++	-

NT, Not tested; −, no detectable toxin; +, low toxin production; ++, intermediate toxin production, +++, strong toxin production.

With the exception of *B. cereus s. s.* Bc14-005, all strains produced less enterotoxin at 37°C, in comparison with that measured at 25°C. *B. thuringiensis* was the only species with detectable Hbl toxin production at 37°C. No enterotoxin production was noted for *B. weihenstephanensis* in either TSBYE or 10% RSM at 37°C; interestingly, although all other representative isolates produced enterotoxin in TSBYE at this temperature, only the *B. cereus s. s.* and *B. thuringiensis* isolates produced detectable toxin in 10% RSM at 37°C.

Overall, toxin production was lowest at 12°C. Neither *B pseudomycoides* nor *B. weihenstephanensis* produced detectable Nhe or Hbl at this temperature; production of Nhe by representative isolates of the other three species was low, and no Hbl production was detected at 12°C.

### *Clostridium perfringens* toxin subtyping

**Figure 4:**
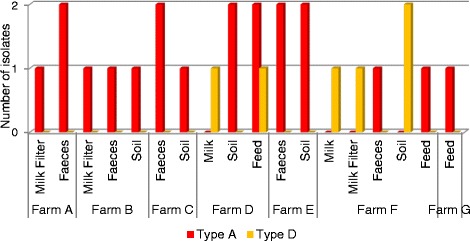
**Distribution of toxin types across all seven farms.** Samples containing *C. perfringens* isolates included: milk filters, raw milk, faeces, soil and animal feed.

Among the 26 isolates, two toxin types were detected - type A and type D (Figure 4); all isolates contained alpha-toxin (type A isolates), whereas only 6 strains had both the alpha and epsilon-toxins (type D). The *Clostridium perfringens* enterotoxin and the beta2 toxin were not detected in any of the isolates. Thus, out of the 26 isolates, 77% were identified as type A and 23% as type D. Type A strains were present on every farm (A to G), whereas type D strains were found only on Farms D and F, which are caprine farms. On Farm D, the type D strains were isolated from feed (grain) and raw milk samples only. On Farm F, the type D strains were isolated from soil, milk and milk filter samples only. No type D strain was isolated from the animal faeces on these farms.

## Discussion

Species of *B. cereus s. l.*, along with *C. perfringens*, may cause illness in both humans and animals. These bacteria are commonly found in many environments, including soil. As such, there is potential for both infection of animals and entry in food chains through contamination of crops, raw milk, or food animals themselves. These organisms are of particular importance in food safety, as their spores may survive thermal treatments, such as pasteurization [16]. Characterization of their populations can thus provide insights into the biology of these organisms, along with associated risks of cross-contamination and infection. This study characterized isolates obtained from seven farms throughout Victoria, Australia, to understand aspects such as population structure, virulence traits, and toxin production.

The most common species identified among *B. cereus s. l.* isolates collected was *B. cereus s. s.*, which comprised 78% of the population. Species diversity across different farm environments was varied; Farms A and E showed the lowest diversity, where only *B. cereus s. s.* was identified; all other farms harboured either two or three species.

Comparative analysis of the growth profiles of representative isolates for each of the 5 species at low temperatures suggested *B. weihenstephanensis* possessed the highest growth rates at all 3 temperatures examined (8°C, 10°C, and 12°C). This characteristic is reflected in the psychrotolerant nature of the organisms, which is known to grow at temperatures as low as 5°C [11,17]. The *B. pseudomycoides* isolate showed the slowest growth rates at 10°C and 12°C, and did not grow at 8°C. Taken together, these results suggest that toxigenic *B. weihenstephanensis* isolates may be of higher risk in cases of contaminated refrigerated food products that have experienced temperature adulteration; conversely, the comparative risk may be lower in the case of *B. pseudomycoides*. Although this data provides an indication of the growth performance of these isolates at low temperature, the duration of lag phase, or the exact maximum growth rate, would require additional bacterial counts in the early growth phase.

PFGE analysis was utilised to examine population diversity among *B. cereus s. l.* isolates. Of the 23 samples where 2 isolates were taken, only 4 contained the same pulsotype (i.e. the pair of isolates from the other 19 samples possessed different pulsotypes). This indicated a high diversity of *B. cereus s. l.* among samples. Indeed, it is probable the actual population diversity of *B. cererus s. l.* in a proportion of the positive samples would have been found to be greater than was identified in this study, had more isolates been taken from those samples. That withstanding, this analysis identified 46 distinct isolates from the original pool of 50.

Two Farm F isolates, Bc13-013 isolated from soil and Bc13-012 isolates from faeces, were indistinguishable by PFGE. Contaminated soil may lead to contamination of the feeding pasture, which in turn may lead to passage to the animal intestinal tract, this may be transient, or lead to colonization of the gut. Either scenario may account for this observation. In the case of the other two non-distinguishable profiles (Bc13-009 and Bc14-016), these were identified in soil samples collected from Farm D in December 2013 and again in March 2014. Both samples were collected from the same pasture field, although not collected at the same area of the field. This suggests a persistent pattern of colonization by this pulsotype in this particular field. Although this pulsotype was not identified in faecal samples from animals on the farm, further insights into its colonization across the farm, and in the herd, would necessitate more extensive sampling. Nonetheless, taken together, both instances where an individual pulsotype was identified in multiple samples highlights the contamination risk associated with resident strains in the farm environment; such bacteria will conceivably be found at higher frequency around the farm environment and in faecal shedding of the herd, and thus at higher risk of contamination of milk, and entry to the dairy food chain.

Further examination of the remaining 42 PFGE profiles indicated a diverse population, not only around individual farm environments, but across the farm environments at large. Each farm had its own distinct community of *B. cereus s. l.* isolates, with no shared PFGE profiles observed between farms; this observation should be contextualised in that the complete population diversity is likely more complex than this study identified. Farms had a minimum of between three and nine pulsotypes among samples taken, as determined by PFGE analysis. *B. cereus s. s.* dominated all farms, comprising a minimum of 63% of species in the case of Farm C, up to 100% of all species identified (Farms A and E). This suggests this species is the dominant member of these *B. cereus s. l.* populations. There was no apparent correlation with farm herd type (i.e. bovine, caprine or ovine) to a higher or lower diversity of isolate pulsotypes or number of species. Since isolation of the organisms does not involve an enrichment step, this does not introduce the possibility of certain isolates out-competing others in the enrichment process, nonetheless possible competition between strains on the Mannitol Egg Yolk Polymyxin Agar (MYPA) medium used for isolation, or indeed the medium itself, may affect their isolation. Certain *B. cereus s. l.* isolates may produce bactericidal toxins that inhibit other isolates [18,19], and this may impact the presence of other isolates in localised environments, or the isolation on solid media such as MYPA. Interestingly, these toxins may be directed at inter-strain and -species competition [19]; which may impart a significant competitive advantage to colonisation of an environment that is potentially the primary reservoir of the organism. Such inhibition was observed among isolates in this study (Additional file 1: Figure S1).

Enterotoxin production by *B. cereus s. l.* isolates is central to the manifestation of clinical illness. The emetic form of illness is attributed, in a large part, to the heat-stable cereulide toxin [20]; in cases of diarrheal illness, two of the important pore-forming toxins involved in this pathogenesis are Hbl and Nhe. Although some evidence suggests Nhe is the key toxin in cases of diarrheal illness [21], it is generally thought that this may not necessarily be true for all strains, and the toxins may in fact play a combined role in disease outcome [22]. To understand the implications of milk contaminated with *B. cereus s. l.*, one representative isolate for each of the species identified on farms was grown in either 10% RSM or TSBYE medium at various temperatures (12°C, 25°C, or 37°C). Interesting, all isolates showed a trend of reduced toxin production in milk, when compared with TSBYE; the extent of which varied depending on the combination of temperature and time measured (24 h or 48 h). This may indicate a reduced risk of toxin production in milk and/or dairy products, relative to other food matrices, and thus for certain forms of *B. cereus s. l.*-induced illness as a result of ingestion of contaminated foodstuffs. Of the five species, the *B. weihenstephanensis* isolate produced the lowest detected Nhe toxin level under all conditions tested, when compared with other isolates. Production of Hbl toxin was lower than that of Nhe under all experimental conditions; indeed Hbl production was only detected for the *B. mycoides* and *B. thuringiensis* isolates. Contrary to this, all isolates produced Nhe. This suggests that all of these isolates may illicit diarrheal disease in human hosts under favourable conditions. Although production of enterotoxins was greatest at 25°C, production of Nhe was identified at 37°C for all isolates, with the exception of *B. weihenstephanensis*. This again implies the capacity of these isolates to cause diarrheal illness in humans, as this reflects the temperature conditions *in vivo*. That withstanding, based on the observation that no enterotoxin was detected in 10% RSM at 37°C, the food vector involved in the infection may play some role in the disease outcome. This may be reflected in the low prevalence of dairy products among foods linked to *B.cereus s. l.* food-poisoning outbreaks [23,24]; however, this does not preclude illness through consumption of contaminated foods, as entertoxin production may also occur in the gut. Toxin production was lowest at 12°C, suggesting that temperature-controlled contaminated food products, such as those refrigerated, may also have a lower associated risk of causing illness. Overall, the *B. cereus s. s.* and *B. thuringiensis* isolates showed highest levels of toxin production across the range of conditions (medium, temperature, time) tested.

Two toxin types were identified among the *C. perfringens* isolates: type A, which may cause foodborne illness in humans [25], however can often be part of the normal intestinal microflora of humans and animals [26]; and type D, which are particularly associated with disease in caprine and ovine animals [27]. Type D isolates were only identified on caprine farms. These results are consistent with the finding of previous studies that showed that *C. perfringens* type D strains are predominantly associated with goats and sheep, rather than cattle [28]. None of the type D isolates identified in this study were found among faecal samples tested (Figure 4), however milk and/or milk filter samples on both farms harbouring type D strains were positive. These results indicate that transmission may have occurred through another route, without intestinal carriage by, or infection of the animals. Type A strains are more ubiquitous and have been found in cattle, as well as caprine and ovine populations [28]. Type A isolates were found on all seven farms in this study, in a diverse range of samples (Figure 4). All type A isolates (along with all type D isolates) did not contain either the *C. perfringens* enterotoxin gene (*cpe*), or the beta2 toxin gene (*cpb2*), as determined by the PCR screening method (data not shown) [29]. Beta2 toxin has been implicated in bovine enteritis cases [28,29], suggesting the isolates in this study may be of lower risk of disease to the herds on the associated farms.

## Conclusion

This study provides insights to the diversity of the *Bacillus cereus* group of bacteria and *Clostridium perfringens* on farm populations in Victoria. First of all, both species were isolated from all seven farms and from the same types of samples. On each farm, the *B. cereus s. l.* population was found to be diverse and distinct to each localised environment. Enterotoxin production in milk was limited compared to broth in the same conditions, suggesting that enterotoxin production by *B. cereus s. l.* may be reduced for dairy products. Type A isolates were dominant among the *C. perfringens* population; however the presence of type D isolates on two of the three caprine farms may be a potential concern with regards to the risk of enterotoxemia in goats.

## Methods

### Bacterial isolates in this study

Isolates were obtained from soil, faeces, feed (grain), milk and milk filter samples from 7 dairy farms across Victoria, Australia (3 bovine, 3 caprine, and 1 ovine). Sampling was performed on 2 separate occasions, in December 2013 and March 2014. Isolation from samples was performed as per Australian standard methods (AS 5013.2-2007 for *B. cereus s. l.*, and AS 5013.16-2006 for *C. perfringens*). This comprised 50 *B. cereus s. l.* and 26 *C. perfringens* isolates. Where possible, two presumptive-positive isolates were taken from each positive sample. Where both rhizoid and non-rhizoid colonies were identified in a positive sample, one of each was taken for further analysis.

### Growth of *B. cereus s. l.* isolates at 8°C and 40°C

Isolates were streaked on in Brain-Heart Infusion (BHI) agar plates and incubated overnight at 30°C. For each isolate, one colony was picked from the BHI plate with an inoculating loop and mixed into 5 ml of BHI broth. After overnight incubation at 30°C the cultures reached a concentration of approximately 10^8^-10^9^ CFU/ml. For growth assessment at 8°C, serial dilutions were prepared and 3 ml cell suspensions were inoculated with the test isolate at a concentration of 10^2^ CFU/ml. Growth was assessed after 14 days by measuring the optical density of the culture at 610 nm. For growth assessment at 40°C, test isolates were streaked on BHI agar plates and incubated at 40°C. Growth was examined after four days. All growth/no growth determinations were repeated in duplicate to confirm the final result.

### Hemolysis testing of *B. cereus s. l.* isolates

Trypticase Soy agar plates with 5% of Sheep Blood were divided in 6 equal sections and inoculated with 24 h BHI broth culture suspensions by touching the surface of the agar with a 1 μl loop. Plates were incubated for 24 h at 35°C and examined for hemolytic activity. Hemolytic strains were identified by a translucent halo of 2 to 4 mm of hemolysis.

### Production of toxin crystals by *B. cereus s. l.* isolates

Analysis of toxin production was carried out based on the FDA standard method, which minimised sequential culturing, and utilises media without curing agents, to reduce the chance of plasmid curing [30]. Nutrient agar slopes were inoculated with a 1 μl loopful of a fresh colony. The slants were incubated for 24 h at 30°C then 4 to 5 days at room temperature, until all the strains sporulated. Smears were prepared with sterile distilled water, air-dried then heat-fixed by passing the slide through the flame of a bunsen burner. The slides were then flooded with methanol. After 30 s the methanol was poured off and the slides air dried. The slides were then flooded with 0.5% basic fuchsin and gently heated from below with a small Bunsen burner until steam was seen. The slides were let to stand for 1 to 2 min before repeating this last step. The slide was then rinsed thoroughly with distilled water, and a cover slip was mounted. The slides were examined for the presence of toxins under 100X magnification using a Leica DM6000B microscope (Leica Microsystems, Germany) with immersion oil.

### Growth kinetics of *B. cereus s. l.* isolates at low temperatures

Isolates were streaked onto BHI agar plates and incubated overnight at 30°C. For each isolate, one colony was picked from the BHI plate with an inoculating loop and mixed into 5 ml of BHI broth. After overnight incubation at 30°C the cultures reached a concentration of approximately 10^8^-10^9^ CFU/ml. After serial dilutions, 3 ml cell suspensions with an approximate concentration of 10^2^ CFU/ml were set in 12-well plates. Sterile BHI broth was used as a negative control and a blank to standardise the data.

The plates were incubated at 8°C, 10°C and 12°C over a period of 14 days. Growth was monitored by taking optical density measurements at 600 nm with a plate-reader spectrophotometer. Growth curves for each strain and temperature were then plotted, using standard curves generated for each isolate to equate absorbance readings to log(CFU/ml) (data not shown). All growth curves and standard curves were measured in triplicate, with each replicate performed on separate occasions.

### PFGE subtyping of *B. cereus s. l.* isolates

An in-house method was developed for PFGE subtyping of *B. cereus s. l.* isolates. Test isolates were streaked on BHI agar plates and incubated for 14–18 h at 30°C. Cell suspensions were then prepared in 2 ml TE buffer (10 mM Tris:1 mM EDTA, pH 8.0), to a final cell optical density of 1.00 at 600 nm, as measured on a spectrophotometer. 400 μl of the cell suspension was mixed with 20 μl of a lysozyme solution (20 mg/ml) and incubated for 2 h at 56°C. Following 1% PFGE-grade agarose (SeaKem Gold; Lonza, Switzerland) was prepared with TE buffer and equilibriated at 55°C. 20 μl of a Proteinase K solution (20 mg/ml) was added to the cell suspensions, followed immediately by 400 μl of the 1% SKG agarose and gently mixed. The plugs were then cast in disposable plug molds (Bio-Rad, UK). After 10 to 15 min at room temperature the plugs were transferred in a Cell Lysis Buffer (50 mM Tris:50 mM EDTA, pH 8.0 + 1% Sarcosyl) containing 25 μl Proteinase K solution (20 mg/ml). The plugs were lysed overnight in a 56°C shaker water bath at 160 rpm. Prior to plug washing, the water bath temperature was reduced to 50°C and the wash solutions were pre-warmed before use. To wash the plugs, the lysis buffer was drained from the tube, and replaced with 5 ml sterile molecular grade water. The tube was then replaced in the water bath at 50°C, and shaken at 160 rpm for 10–15 min. One more wash with molecular grade water was repeated as before, followed by four washes with TE buffer. Plugs were then transferred to 1.5 ml centrifuge tubes filled with 1 ml sterile TE buffer and stored at 4°C until used.

For PFGE analysis, DNA plugs were restricted using either *Sbf*I or *Not*I endonucleases (New England Biolabs, US). For both enzymes, 40U was incubated into a 200 μl reaction mix at 37°C for 2 h. After restriction, the plugs were equilibrated for 10–15 min in 0.5X Tris Borate EDTA buffer (40 mM Tris–HCl: 45 mM boric acid: 1 mM EDTA ) at room temperature.

DNA fragments were separated on a 1% SeaKem Gold agarose gel using a CHEF-Mapper (Bio-Rad, UK) with the following program: auto algorithm; low molecular weight = 20 kb; high molecular weight = 700 kb; select default values except where noted by pressing “enter”; run time = 21 h; initial switch time = 5 s; final switch time = 80 s; tank temperature = 14°C. The *Saccharomyces cerevisiae* CHEF DNA Size Marker (Bio-Rad, UK), already restricted, and *Salmonella* ser. Braenderup H9812 chromosome, prepared according to the Standard Operating procedure for PusleNet PFGE for *Salmonella* [31] and restricted with 50 U *Xba*I restriction endonuclease (New England Biolabs, US), were used simultaneously as DNA-size standards for analysis. Gels were observed after staining 30 min in a 300 ml 1% GelRed (Biotium, US) solution and de-staining for 40 min in a 300 ml distilled water bath. Cluster analysis was performed with BioNumerics v6.5 software (Applied Maths, Belgium). Similarity matrixes were produced using the Dice coefficient for the two experiments (digestion with *Not*I and *Sbf*I), then a combined analysis was performed using the unweighted pair group method with arithmetic mean (UPGMA), with optimisation and tolerance settings of 1%.

### Semi-quantitative toxin production assay

The production of the enterotoxins Nhe and Hbl was investigated with five isolates, representing each of the *Bacillus cereus s. l.* species isolated in this study: *B. cereus s. s.* (Bc014-005), *B. pseudomycoides* (Bc14-006), *B mycoides* (Bc14-009), *B. weihenstephanensis* (Bc14-026), and *B. thuringiensis* (Bc13-016). Toxin production was measured at three temperatures: 12°C, measured at 48 h; and 25°C and 37°C, both measured after 24 h and 48 h. Toxin production was assayed in two test media: 10% reconstituted skim milk (RSM) or tryptic soy broth with yeast extract (TSBYE), separately for each representative isolate. Overnight cultures grown at 30°C were diluted to a concentration of approximately 10^6^ CFU/ml in the test medium. A final concentration of 10^4^ CFU/ml in 10 ml of the test medium was used for each test. The tubes were incubated without agitation. The Nhe and Hbl toxins were detected using the Duopath *Cereus* Enterotoxins detection kit (Merck, US). For 10% RSM cultures, 100 μl was added to 900 μl sterile molecular grade water; for TSBYE broth cultures, 100 μl of sample was added to 100 μl 10% RSM in 800 μl sterile molecular grade water. A volume of 150 μl was the applied to the toxin test chip and results were assessed after 30 min, according to the product datasheet. The intensity of the line was scored as – (no line), + (faint line), ++ (clear line), +++ (thick clear line), to achieve a semi-quantitative measure of toxin production.

### Toxin subtyping of *C. perfringens* isolates

DNA extraction was achieved by picking up half a colony from fresh BHI cultures and mixing it in 200 μl molecular biology grade sterile water. After heating at 95°C for 15 min, the samples were centrifuged for 10 s at 14,000 rpm. The PCR was performed with a FTS-960 Thermal Sequencer (Corbett Life Science, US), following the protocol described by Kalender et al. [32]. Representative data set is shown in Additional file 2: Figure S2.

## Additional files

Additional file 1: Figure S1. Competitive inhibition of *B. cereus s. l.* species. *B. cereus s. s.* (Bc14-005) inhibiting a *B. pseudomycoides* (Bc14-006) isolate. Additional file 2: Figure S2.
*C. perfringens* toxin type PCR. Example of the results obtained for the *C. perfringens* toxin type PCR assay. Lane 1, 100 bp ladder (Bioline, Australia); Lane 2, Cp13-014 (type A); Lane 3, Cp14-014 (type D); Lane 4, ATCC 12916 (type A, *cpe* positive, control strain); Lane 5, 7581 (type D, control strain), Lane 6, 100 bp ladder (Bioline, Australia). Numbers listed on the left side indicate DNA marker band sizes. Arrows on the right side refer to gene products in lanes 2-5 (enterotoxin, *cpe*; epsilon toxin, *etx*; alpha toxin, *cpa*).
