# Supplementary material for: Characterization of the spore-forming Bacillus cereus sensu lato group and Clostridium perfringens bacteria isolated from the Australian dairy farm environment
Source: BMC Microbiol. 2015 Feb 19;15:38. doi: 10.1186/s12866-015-0377-9 (PMC4336692; doi:10.1186/s12866-015-0377-9)
Supplement: Additional file 1: Figure S1. — Competitive inhibition of B. cereus s. l. species. B. cereus s. s. (Bc14-005) inhibiting a B. pseudomycoides (Bc14-006) isolate. [file 12866_2015_377_MOESM1_ESM.docx]

**Supplementary Figure 1 Competitive inhibition of *B. cereus s. l.* species.** *B. cereus s. s.* (Bc14-005) inhibiting a *B. pseudomycoides* (Bc14-006) isolate.
