# Supplementary material for: Characterization of the spore-forming Bacillus cereus sensu lato group and Clostridium perfringens bacteria isolated from the Australian dairy farm environment
Source: BMC Microbiol. 2015 Feb 19;15:38. doi: 10.1186/s12866-015-0377-9 (PMC4336692; doi:10.1186/s12866-015-0377-9)
Supplement: Additional file 2: Figure S2. — C. perfringens toxin type PCR. Example of the results obtained for the C. perfringens toxin type PCR assay. Lane 1, 100 bp ladder (Bioline, Australia); Lane 2, Cp13-014 (type A); Lane 3, Cp14-014 (type D); Lane 4, ATCC 12916 (type A, cpe positive, control strain); Lane 5, 7581 (type D, control strain), Lane 6, 100 bp ladder (Bioline, Australia). Numbers listed on the left side indicate DNA marker band sizes. Arrows on the right side refer to gene products in lanes 2-5 (enterotoxin, cpe; epsilon toxin, etx; alpha toxin, cpa). [file 12866_2015_377_MOESM2_ESM.docx]

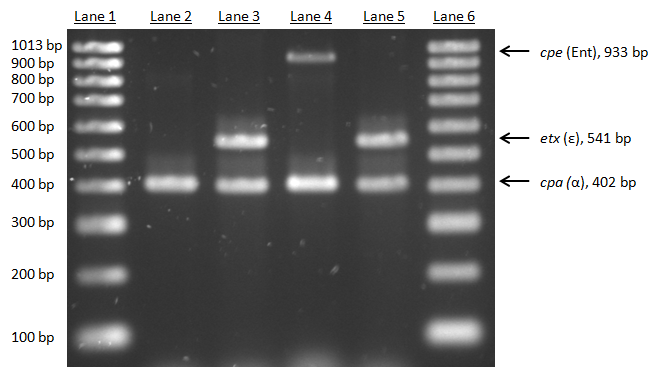


**Supplementary Figure 2 *C. perfringens* toxin type PCR.** Example of the results obtained for the *C. perfringens* toxin type PCR assay. Lane 1, 100bp ladder (Bioline, Australia); Lane 2, Cp13-014 (type A); Lane 3, Cp14-014 (type D); Lane 4, ATCC 12916 (type A, *cpe* positive, control strain); Lane 5, 7581 (type D, control strain), Lane 6, 100bp ladder (Bioline, Australia). Numbers listed on the left side indicate DNA marker band sizes. Arrows on the right side refer to gene products in lanes 2-5 (enterotoxin, *cpe*; epsilon toxin, *etx*; alpha toxin, *cpa*).
